# Identifying and applying a highly selective probe to simultaneously determine the *O*-glucuronidation activity of human UGT1A3 and UGT1A4

**DOI:** 10.1038/srep09627

**Published:** 2015-04-17

**Authors:** Li Jiang, Si-Cheng Liang, Chao Wang, Guang-Bo Ge, Xiao-Kui Huo, Xiao-Yi Qi, Sa Deng, Ke-Xin Liu, Xiao-Chi Ma

**Affiliations:** 1 College of Pharmacy, Key Laboratory of Pharmacokinetic and Drug Transport of Liaoning, Academy of Integrative Medicine, Dalian Medical University, Dalian, 116044, China; 2 Laboratory of Pharmaceutical Resource Discovery, Dalian Institute of Chemical Physics, Chinese Academy of Sciences, Dalian, China; 3 Graduate School of Chinese Academy of Sciences, Beijing, China; 4 Second Affiliated Hospital of Dalian Medical University, Dalian, China

## Abstract

Glucuronidation mediated by uridine 5′-diphospho (UDP)-glucuronosyltransferase is an important detoxification pathway. However, identifying a selective probe of UDP- glucuronosyltransferase is complicated because of the significant overlapping substrate specificity displayed by the enzyme. In this paper, desacetylcinobufagin (DACB) 3-*O*- and 16-*O*-glucuronidation were found to be isoform-specific probe reactions for UGT1A4 and UGT1A3, respectively. DACB was well characterized as a probe for simultaneously determining the catalytic activities of *O*-glucuronidation mediated by UGT1A3 and UGT1A4 from various enzyme sources, through a sensitive analysis method.

Uridine 5′-diphospho (UDP)-glucuronosyltransferases (UGTs), an important superfamily of membrane-bound enzymes comprising three subfamilies (UGT1A, UGT2A and UGT2B), play a vital role in the metabolic elimination and detoxification of endobiotics and xenobiotics via glucuronidation^1^,^2^,^3^,^4^,^5^. However, determining the activities of various UGTs is a great challenge, due to their complex overlapping substrate activities and broad substrate specificities^6^,^7^. Additionally, no probe substrate is qualified to distinguish the two UGT isoforms (which have a high homology) in a single measurement. Therefore, developing highly selective probes for UGTs is necessary to measure the catalytic activities from different enzyme sources.

Among the human UGT isoforms, UGT1A3 and UGT1A4 are recognized to be important and to account for 9% and 26% of the total UGT-catalyzed conjugation reactions, respectively^8^. However, UGT1A3 and UGT1A4 are encoded by genes that share 93% homology, which results in their broad and overlapping substrate specificities, notably in the *O*-glucuronidation reaction^9^,^10^. UGT1A3 and UGT1A4 are frequently involved in the glucuronidation of amines^9^, carboxylic acids^10^, steroids^10^ and opioids^11^. These homologs share numerous substrates including many commercially available drugs such as clozapine^9^, amitriptyline^12^,^13^, cyproheptadine^12^,^13^, clomipramine^12^,^13^, and steroid hormones^14^,^15^. Therefore, considering the high degree of substrate overlap between these two isoforms, accurate assessment of the bioactivities of UGT1A3 and UGT1A4 is quite necessary. Additionally, the activities of UGT1A3 and UGT1A4 also show up to 60-fold variation between individuals^16^, increasing clinical risks such as significant variations of plasma concentrations and subsequent toxicity. Therefore, measuring the individual contributions of UGT1A3 and UGT1A4 will assist clinical dosage regulation in personalized medicine, especially when the metabolism of oral drugs is mainly mediated by UGT1A3 and UGT1A4.

Currently, chenodeoxycholic acid (CDCA) and vitamin D_3_ (VD_3_) are used as UGT1A3 probes^17^,^18^,^19^. Additionally, trifluoperazine (TFP) can be used as a probe for UGT1A4^20^. However, the selectivities of CDCA and VD_3_ are limited as UGT1A3 probes. In the case of CDCA, UGT1A1, UGT1A3 and UGT1A8 isoforms are all involved in its *O*-glucuronidation^17^. Similarly, F6-1, 23S, 25(OH)_3_D_3_ (a trace metabolite of VD_3_) can be used as a UGT1A3 probe only by a semi-quantitative method, and UGT2B4 and UGT2B7 are also partly involved in the reaction of the probe with UGT1A3^18^. For UGT1A4, TFP can be used as a specific probe only for measuring *N*-glucuronidation, not for *O*-glucuronidation^20^. Therefore, developing a novel probe is necessary to establish a highly specific determination of the *O*-glucuronidation activities mediated by UGT1A3 and UGT1A4 individually.

Bufadienolides with their unique skeleton characteristics as C24 steroids, are rich natural resources in Chinese medicine “Chansu”^21^. In our previous research, many novel bufadienolide derivatives were obtained by microbial transformation and chemical synthesis in order to develop several new leading compounds with potentially biological application^22^,^23^,^24^,^25^,^26^. The previous investigation also reported that some bufadienolides, such as marinobufagin can be metabolized to form 3-*O*- glucuronide in pool human liver microsomes (HLMs)^27^. Additionally, our preliminary screening results demonstrated that several bufadienolides can be glucuronidated in HLMs and human intestinal microsomes (HIMs), notably at the C-3 and C-16 positions. These findings prompted us to screen bufadienolide analogues for measuring the activities of various UGTs in biological samples.

After screening a series of natural and transformed bufadienolides (Figure S1) using human UGT isoforms in the present paper, we found an isoform-specific probe substrate (desacetylcinobufagin, DACB, Figure 1) for simultaneously determining the *O*-glucuronidation activities of UGT1A3 and UGT1A4 in the different enzyme resources. The selectivities for UGTs 1A3 and 1A4 were determined by chemical inhibition testings, screening assays with human UGT isoforms, and the correlation assays. Our results indicated that UGT1A4 catalyzed 3-*O*-glucuronidation of DACB with excellent selectivity, and UGT1A3 was found to dominantly catalyze the 16-*O*-glucuronidation of DACB, based on the kinetic studies of HLMs and UGT isoforms. It is firstly reported the simultaneous and specific determination of *O*- glucuronidation activities of UGT1A3 and UGT1A4.

## Results

### Identification of glucuronidation metabolites

Incubation of DACB with HLMs in the presence of uridine 5′-diphospho-glucuronic acid (UDPGA) yielded two glucuronide metabolites (M-1 and M-2). The formation of M-1 and M-2 was dependent on time, microsome concentrations, and UDPGA. These two metabolites were biosynthesized and isolated through chromatographic methods. M-1 as a white powder in MeOH, was prepared by biotransformation using HLMs. The electrospray ionization-mass spectrometry (ESI-MS) of M-1 showed an [M-H]^−^ ion peak at *m/z* 574.2 in the negative-ion mode with a characteristic *m/z* 176, indicating a molecular formula of C_30_H_39_O_11_. Compared with desacetylcinobufagin (DACB), six additional oxygen-bearing carbon signals were observed at *δ* 101.5, 71.5, 76.1, 73.2, 75.6 and 170.4, indicating that M-1 was a monoglucuronide metabolite. The carbon signal of C-3 shifted up to *δ* 73.4. In the HMBC spectrum, H-1′ (*δ* 4.26) had a key long-range correlation with C-3 (*δ* 73.4), suggesting that the glucuronosyl group should be at 3-OH. Therefore, M-1 was identified as desacetylcinobufagin-3*β*-glucuronate. Similarly, the ESI-MS of M-2 provided a [M-H]^−^ ion peak at *m/z* 574.3, suggesting its molecular formula of C_30_H_39_O_11_. In the ^13^C-NMR spectrum, the additional carbon signals of *δ*101.9, 71.2, 75.9, 73.0, 75.6 and 170.3 indicated that M-2 was also a monoglucuronide metabolite of DACB. The carbon signal of C-16 shifted up to *δ* 76.9, due to aglycosidation shift. Additionally, C-16 had the HMBC correlations with H-15 and H-17, and C-1′ had a key correlation with H-16. In combination, these results suggested that the glucuronosyl substitution should be at 16-OH. Therefore, the structure of M-2 was characterized as desacetylcinobufagin-16*β*-glucuronate. All of the ^1^H- and ^13^C-NMR spectral data were unambiguously assigned by 2D-NMR spectra (Table S1 and Figs. S2–3).

### Preparation of DACB metabolites (M-1 and M-2) by various animal liver microsomes

To enable wide application of DACB as a selective probe in the future, the preparative methods for DACB and its glucouronidation metabolites were established. After comparing the metabolism of DACB in various liver microsomes (see the Methods section), RLMs (rabbit liver microsomes) exhibited greater production of M-1, whereas MLMs (monkey liver microsomes) were the preferred model for the large-scale preparation of M-2 (Fig. S4). In addition, the preparative method for DACB by microbial transformation was also described in our previous report^17^.

### UGT isoforms involved in DACB glucuronidation

**Figure 2:**
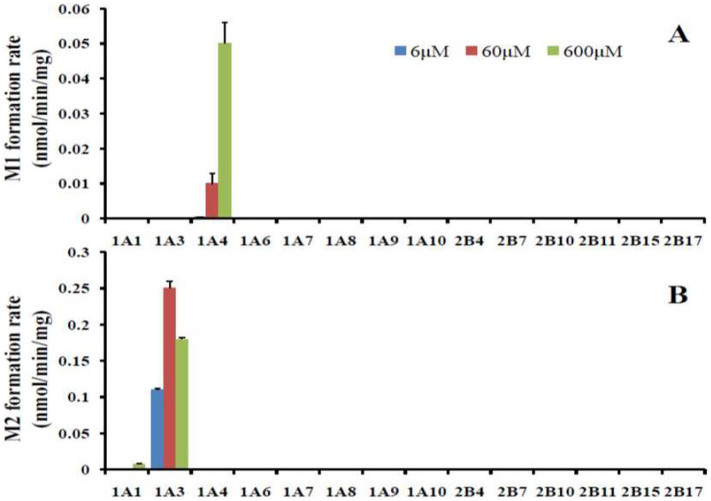
Isoform specificity of 3*β*- (A) and 16*β*-*O*-glucuronidation (B).

DACB glucuronidation by recombinant UGT Supersomes™ was investigated using a panel of fourteen recombinant UGT isozymes (1A1, 1A3, 1A4, 1A6, 1A7, 1A8, 1A9, 1A10, 2B4, 2B7, 2B10, 2B11, 2B15 and 2B17). Surprisingly, UGT1A4 mainly dominated the formation of M-1 at three different substrate concentrations (6, 60 and 600 μM) with a high selectivity (Fig. 2A). Additionally, Fig. 2B indicated that UGT1A3 had a good selectivity in promoting M-2 formation. Only UGT1A1 displayed the limited ability to generate M-2 at the highest substrate concentration (600 μM), which was 30 times less than UGT1A3 (Fig. S5). Up to now, DACB is the most selective probe for *O*-glucuronidation simultaneously mediated by UGT1A3 and UGT1A4.

### Chemical inhibition

**Figure 3:**
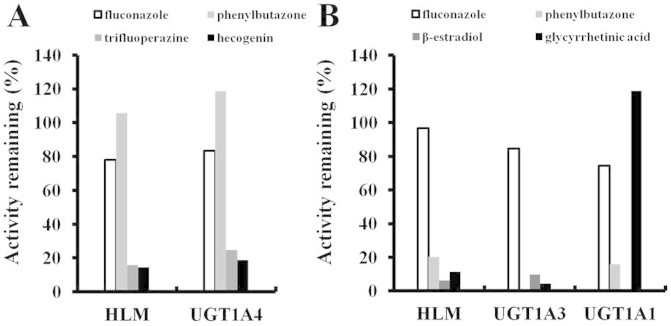
Inhibition assay of 3*β*- (A) and 16*β*-*O*-glucuronidation (B) by UGT inhibitors in HLMs.

To confirm the key roles of UGT1A3 and UGT1A4 in the 16-*O*- and 3-*O*-glucuronidation of DACB, respectively, chemical inhibition studies were also performed. As shown in Fig. 3, phenylbutazone^20^, TFP^20^, fluconazole^28^ and hecogenin^29^ were used to inhibit the 3-*O*-glucuronidation of DACB mediated by UGT1A4. Our results showed that TFP (a specific substrate of UGT1A4) and hecogenin (a specific inhibitor of UGT1A4) can significantly inhibit the 3-*O*-glucuronidation of DACB. The similar inhibitory concentration for 50% reduction (IC_50_) values of hecogenin for UGT1A4 and HLMs also strongly suggested that UGT1A4 can catalyze the 3-*O*-glucuronidation of DACB with satisfactory selectivity (Figs. S6A and B). Similarly, the formation of M-2 (16-*O*-glucuronate) was significantly inhibited by phenylbutazone^20^, glycyrrhetinic acid^30^ and *β*- estradiol^31^ at substrate concentration of 60 μM. Additionally, the equal IC_50_ values for glycyrrhetinic acid (a selective inhibitor of UGT1A3), UGT1A3 and HLMs were observed for 16-*O*-glucuronidation (Figs. S6C and D). This evidence strongly indicated that the 16-*O*-glucuronidation of DACB is selectively catalyzed by UGT1A3.

### Enzyme kinetics analysis with UGT1A3, 1A4 and HLMs

**Figure 4:**
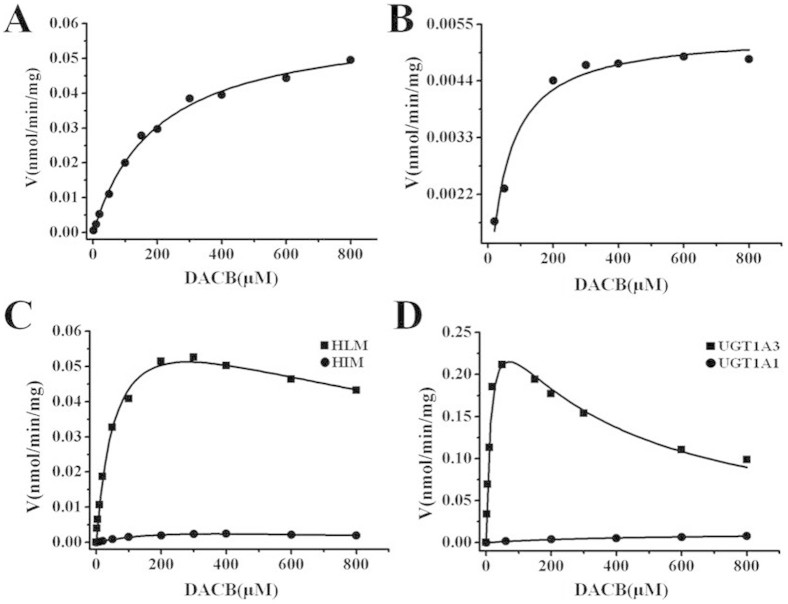
The enzyme kinetics of DACB glucuronidation at C-3 or C-16 in HLMs and UGT isoforms. (A) 3-*O*-glucuronidation of DACB in HLMs; (B) 3-*O*- glucuronidation of DACB in UGT1A4; (C) 16-*O*-glucuronidation of DACB in HLMs and HIMs; (D) 16-*O*-glucuronidation of DACB in UGT1A3 and UGT1A1.

To further characterize the isoform-specific biocatalysis by UGT1A3 and UGT1A4, kinetic analyses were performed for HLMs, HIMs, recombinant UGT1A3 and UGT1A4, respectively (Fig. 4). With respect to M-1 formation (3-*O*-glucuronidation), similar Michaelis-Menten kinetics were observed in HLMs and UGT1A4, respectively, as evidenced by the Eadie-Hofstee plots (Fig. S7). Additionally, M-1 formation in pooled HLMs showed a similar K_m_ value relative to UGT1A4 (Table 1), suggesting that UGT1A4 was primarily responsible for the 3-*O*-glucuronidation of DACB. For M-2 formation (16-*O*-glucuronidation), the substrate inhibition kinetics were observed for HLMs and UGT1A3, respectively (Figs. 4C–D and S7). Although the isoform screening experiment suggested that UGT1A1 was also partly involved in M-2 formation at a high substrate concentration (600 μM), it exhibited very low glucuronosyltransferases activity and enzyme affinity. The affinity and clearance (V_max_/K_m_) of UGT1A1 in 16-*O*-glucuronidation (M-2) were less than 30 and 825 fold greater, respectively, compared with UGT1A3. These findings further showed that DACB could serve as an ideal probe to simultaneously measure the *O*-glucuronidation activities of UGT1A3 and UGT1A4 in complex enzyme systems.

### Expression-activity correlation of UGT1A3, 1A4 in HLMs

**Figure 5:**
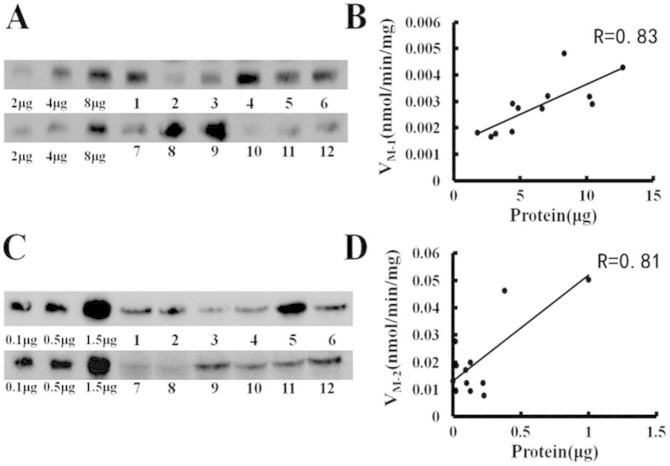
The correlation analysis between the expression of UGT isoforms and the DACB glucuronidation rate in individual HLMs. (A) Western blots of UGT1A3 in individual HLMs; (B) the correlation between UGT1A3 expression and DACB 16-*O*-glucuronidation rates in 12 individual HLMs; (C) Western blots of UGT1A4 in individual HLMs; (D) the correlation between UGT1A4 expression and DACB 3-*O*-glucuronidation rates in 12 individual HLMs.

To evaluate the metabolic activities of UGT1A3 and UGT1A4 in HLMs accurately, the glucuronidation activities of UGT1A3 and UGT1A4 in a panel of HLMs from 12 individuals were determined using the formation of M-1 and M-2, respectively. Additionally, the expression levels of UGT1A3 and UGT1A4 in these individual HLMs were measured using western blot technique. The correlation analysis of DACB 3-*O*- and 16-*O*-glucuronidation rates with the expression levels of UGT1A3 and UGT1A4 in the corresponding individual HLMs, showed strong correlations with the correlation coefficients (R) in the range of 0.79 to 0.82 (P < 0.05) (Fig. 5). Our results demonstrated that DACB is a highly selective probe to simultaneously measure the *O*-glucuronidation bioactivities of UGT1A3 and UGT1A4 in various biological samples.

### Application of DACB as the selective probe to simultaneously determine the activities of UGT1A3 and UGT1A4

**Figure 6:**
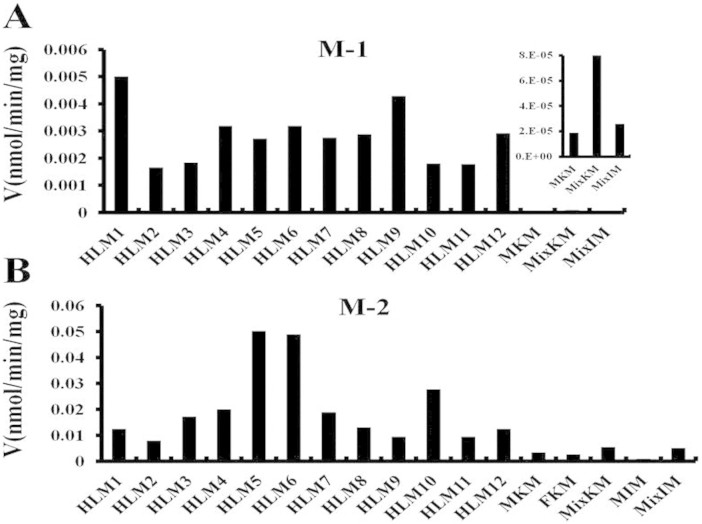
3*β*- (A) and 16*β*-O-glucuronidation (B) in 12 individual HLMs and organ microsomes including the kidney and intestine from different genders.

To widely apply DACB in measuring the real *O*-glucuronidation bioactivities of UGT1A3 and UGT1A4 in various biological samples, we had established a sensitive method using LC-MS/MS (Fig. S8). After optimizing the pH values of various incubation systems, we found that pH values in the range of 7–9 were best for determining the bioactivities of UGT1A3 and UGT1A4 (Fig. S9). In using DACB as a probe, significant *O*-glucuronidation bioactivity differences between UGT1A3 and UGT1A4 in individual human livers (Fig. 6) were elucidated for the first time. And we also revealed that the activity of UGT1A4 in mediating *O*- and *N*-glucuronidation had a positive correlation in HLMs (Fig. S10), which is important for further clarifying the stereochemical structure and catalytic mechanism of UGT1A4. Additionally, animal species differences for UGT1A3 and UGT1A4 were analyzed using DACB. The UGT metabolic profiles indicate that the 16-*O*-glucuronidation of DACB can occur in all species except RtLMs, whereas 3-*O*- glucuronidation occurs in DLMs, MLMs, MsLMs, RLMs and TLMs. Kinetic studies using DACB as a probe for UGT1A3 and UGT1A4 were performed to compare differences between species (Table S2). For DACB-3-*O*-glucuronidation (M-1) mediated by UGT1A4, PLMs, RLMs and MLMs exhibited Michaelis–Menten kinetics, whereas DLMs showed biphasic kinetics (Fig. S11). According to the *CL_int_* values, the activities of UGT1A4 in different animal species were order as Rabbit > Monkey > Mouse > Dog > Pig for M-1 formation. Similarly, the formation of DACB-16-*O*-glucuronidation (M-2) was selectively mediated by UGT1A3 following Michaelis–Menten kinetics in PLMs, DLMs and MLMs, while substrate inhibition kinetics was displayed in RLMs (Fig. S12). Additionally, the *CL_int_* activities of UGT1A3 were MLM > RLM > PLM > DLM (Table S2). These results for the UGT1A3 and UGT1A4 activities, as measuring by using DACB as a selective probe, may provide important guidance for the rational selection of model animals in preclinical studies of new drugs.

In conclusion, through a systematic screening of bufadienolide derivatives, DACB is found to be a highly selective probe substrate for UGT1A3 and UGT1A4, two important drug-metabolizing isoforms in humans that have substantial substrate overlap. DACB 3-*O*-glucurondiation could serve as the first probe for determining the *O*-glucuronidation activity of UGT1A4, and DACB 16-*O*-glucurondiation can serve as a specific probe for the activity of UGT1A3. Additionally, DACB and its metabolites are easily prepared, and a sensitive and rapid analysis method is established by using LC-MS/MS. Notably, DACB as a highly selective probe, is now reported to distinguish the *O*-glucuronidation of UGT1A3 and UGT1A4 proteins which share 93% homology. Furthermore, we can apply this novel probe substrate to characterize the bioactivities of UGT1A3 and UGT1A4 in different biological samples, describing their differences in individual human livers and samples from various animal species. Our findings strongly indicate that this isoform-specific probe can clearly and simultaneously distinguish the *O*-glucuronidation functions of UGT1A3 and UGT1A4 in various biological samples and evaluate the variation in UGT1A3 and UGT1A4, due to the influence of genetic and environmental factors.

## Methods

### Chemicals and reagents

Alamethicin, Brij 58, magnesium chloride, D-saccharic acid 1,4-lactone, β-glucuronidase, UDP-glucuronic acid trisodium salt (UDPGA), phenylbutazone, fluconazole, β-estradiol, glycyrrhetinic acid were purchased from Sigma-Aldrich (St. Louis, MO). Desacetylcinobufagin (DACB) was isolated from Chansu and identified by NMR and ESI mass spectrometry as described previously. The purity was greater than 98% as determined by high-performance liquid chromatography-ultraviolet spectroscopy (HPLC-UV). Liver microsomes from humans, rats (RtLMs), Rabbits (RLMs), mice (MsLMs), dogs (DLMs), monkeys (MLMs), pigs (PLMs) as well as human intestine (HIMs) were purchased from Rild Research Institute for Liver Diseases (Shanghai, China). A panel of 12 commercial recombinant human UGT isoforms (UGT1A1, -1A3, -1A4, -1A6, -1A7, -1A8, -1A9, -1A10, -2B4, -2B7, -2B10, -2B15 and -2B17) were purchased from BD Gentest (Woburn, USA). UGT2B11 was provided by Prof. B.J. Wu (Jinan University, Guangdong, China). All bufadienolides (compounds **1–16**) used for the screening tests were isolated and prepared from crude Chansu materials, the microbial transformation of natural bufadienolides or from biological samples isolated from rats after the administration of Chansu by the authors (X.C. Ma, C. Wang and J. Ning). The chemical structures of the bufadienolides were unambiguously identified using NMR and MS techniques; and their purities were above 98% as determined by HPLC-diode array detector (DAD) analysis. All other reagents including methanol, acetonitrile, hydrochloric acid, dimethylsulfoxide (DMSO), formic acid and trifluoroacetic acid were either of HPLC grade or of the highest commercially available grade.

### Incubation system

The standard incubation system for the UGT reaction included HLMs (5 mg of protein/mL), UDPGA (40 mM), Tris-HCl buffer (pH 7.4), MgCl_2_ (50 mM), 25 μg/mL alamethicin, and substrates in a final volume of 200 μL. The volume of organic solvent was less than 1%. After 60 min of incubation at 37°C, the reaction was terminated by adding 0.1 mL of methanol. The sample was then centrifuged at 20,000 *g* for 20 minutes to obtain the supernatant for LC-UV-ESI analysis. Control incubations without UDPGA, without substrate, without microsomes were performed to ensure that the metabolites produced were microsome- and UDPGA-dependent.

### LC-MS Assay

The Agilent 1200 HPLC system consisted of a quaternary delivery system, a degasser, an auto-sampler and a UV-detector. The chromatograph was equipped with an Elite SinoChorm Ocean Data Standards-Best Practices (2.1 × 150 mm, 5 μM) analytical column. The mobile phase consisted of an acetonitrile-0.1% formic acid aqueous solution at a flow rate of 0.5 mL/min. An Applied Biosystems MDS Sciex API 3200 Triple Quadrupole Mass Spectrometer (MS/MS) equipped with an electrospray ionization (ESI) source was used to analyze potential metabolites, and the system was operated in the negative mode M1 (575.0 → 575.0) and M2 (575.0 → 381.0). The optimized ion spray voltage and temperature were set at 5,000 V and 600°C, respectively. The curtain gas (CUR) is set at 10 L/min; gas1 and gas 2 (nitrogen) were set at 45 and 40 psi, respectively, and the dwell times were 150 ms. Nitrogen was used as the curtain gas and collision gas, controlled at 13 and 6 psi, respectively. The quantification assay was performed using multiple reaction monitoring.

### Metabolite biosynthesis and NMR spectrometry

The glucuronidation metabolites (M-1 and M-2) of DACB were biosynthesized and purified for structure elucidation and quantitative analysis. The enzymatic biosynthesis of M-1 and M-2 was conducted using RLMs and MLMs, respectively, because they can efficiently catalyze the formation of each metabolite detected in other microsomal samples. In brief, 40 mM DACB was incubated with RLMs/MLMs (5 mg/mL), 50 mM Tris-HCl buffer, 50 mM MgCl_2_, and 40 mM UDPGA in 1 mL of the mixtures for 8 h. The stock solution of DACB (80 mM) was prepared in methanol. The concentration of organic solvent in the final incubation was 1%. The reaction is terminated by adding 0.5 mL of methanol. After removing the protein by centrifugation at 20,000 g for 20 min at 4°C, the combined supernatants were loaded onto a solid-phase extraction cartridge (C_18_, 1000 mg; Agela Technologies Inc., Newark, DE), which was preconditioned by sequential washing with 5 mL of methanol and 5 mL of water containing 0.2% formic acid. After loading of the incubation material, the cartridge was washed with 15 mL of water containing 0.2% formic acid. Then, the trapped compounds were eluted with 5 mL of methanol and blown dry with nitrogen gas at 20°C. Finally, the residual was redissolved in 1 mL of methanol and separated by HPLC (Agilent 1200) equipped with a quaternary delivery system, a degasser, an auto-sampler, a UV-detector and a Thermo hemi-preparation ODS (10 × 250 mm, 5 μm). The mobile phase consisted of acetonitrile (A)-0.3‰ trifluoroacetic acid aqueous solution (B) at a flow rate of 1.5 mL/min with a linear gradient from initially 15% to 90% A over 15 min. The fractions containing M-1 and M-2 were collected and dried *in vacuo*. The purities of M-1 and M-2 were approximately 98% by HPLC-UV analysis. The structures of the metabolites were determined by spectral methods including ^1^H-, ^13^C-NMR, HSQC and HMBC. All of the experiments were recorded on a Bruker AV-600 (Bruker, Newark, Germany). The purified metabolites are stored at −20°C before being dissolving in MeOD-*d_4_* (Euriso-Top, Saint-Aubin, France) for the NMR analysis. The key HMBC correlations of M-1 and M-2 were used to identify the sites of glucuronic acid conjugation in their chemical structures.

### Assay with recombinant UGTs

DACB glucuronidation was measured in reaction mixtures containing recombinant human UGT1A1, 1A3, 1A4, 1A6, 1A7, 1A8, 1A9, 1A10, 2B4, 2B7, 2B10, 2B11, 2B15 and 2B17. The incubations were performed using the standard incubation system. Three substrate concentrations (6, 60 and 600 μM) were used in this study: 60 and 600 μM were the approximate concentrations at the V_max_ and K_m_ values for HLMs, respectively; 6 μM was used to evaluate the catalytic activity of the UGT isoforms with a high affinity for DACB glucuronidation. All of the assays were conducted at 37°C for 60 min with a final protein concentration of 0.5 mg of protein/mL. HPLC-UV-MS/MS was used to monitor the produced metabolites.

### Correlation study

A correlation analysis between the expressed level of UGT1A3 and UGT1A4 protein and the glucuronidation rates of DACB in 12 HLMs was performed. The quantitative analysis of UGT1A3 and UGT1A4 was performed using western blot analysis. Briefly, 10 μg of each of 12 HLMs was separated on 10% sodium dodecyl sulfate (SDS)-polyacrylamide gel and then transferred electrophoretically to either a polyvinylidene difluoride (PVDF) or nitrocellulose membrane. The PVDF membrane, Immobilon-P (Millipore Corporation, Billerica, MA), was probed with an anti-human UGT1A3 antibody. The quantitative analysis was performed using the Image Quant TL Image Analysis software (GE healthcare). The correlation analysis for the HLMs from the 12 donors was determined using Spearman's rank method. When the value was greater than or equal to 0.5 and the P value was less than 0.005, the correlations were considered statistically significant. Additionally, TFP (a selective probe for UGT1A4 *N*-glucuronidation) was used in this correlation study of *N*- and *O*-glucuronidation mediated by UGT1A4. HLMs from 12 individuals were used in this study. The 3-*O*-glucuronidation rate of DACB was compared with the rate with TFP in these 12 HLMs *via* linear regression. The concentrations of DACB and TFP were 60 and 100 μM, respectively, which was similar to their *K*_m_ values; the protein concentration of the HLMs was 0.5 mg/mL, and the reaction time was 30 min.

### Chemical inhibition study

DACB was incubated with HLMs in the presence or absence of the following various UGT-specific inhibitors: the UGT1A inhibitor phenylbutazone (500 μM)^20^, the UGT1A4 specific substrate TFP^20^, the UGT 2B7 inhibitor fluconazole (2.5 mM)^28^, the UGT1A4 specific inhibitor hecogenin^29^, the UGT1A3 inhibitor glycyrrhetinic acid (50 μM)^30^ as well as the UGT1A1 inhibitor *β*-estradiol (100 μM)^31^. DACB (60 μM) was incubated with HLMs, UGT1A1 and UGT1A3, respectively, in the presence or absence of these inhibitors. To further assess the role of UGT1A3, the inhibition of glycyrrhetinic acid (0–60 μM) for DACB glucuronidation catalyzed by HLMs and UGT1A3 was investigated. All of the incubations were performed for 60 min using HLMs with a concentration of 0.3 mg of protein/mL, 0.05 mg of protein/mL for UGT1A3 and 0.25 mg of protein/mL for UGT1A4. Similarly, for the UGT1A4 isoform, hecogenin (1–80 μM) was used as a specific inhibitor to determine the differences of inhibition effects between HLMs and UGT1A4. The IC_50_ value representing the concentration that inhibited 50% of the control activity was determined as described previously.

### Kinetics study

To estimate the kinetic parameters, DACB (1–800 μM) was incubated with multiple sources of pooled microsomes (HLMs, HIMs, PLMs, RLMs, MLMs and DLMs) or recombinant human UGT1A1, UGT1A3 and UGT1A4. To ensure that less than 10% of the substrate was metabolized in all incubations, different incubation times and protein concentration conditions were selected with 60 min of incubation and 0.15 to 0.75 mg of protein, to confirm the linear interval of metabolite turnover rates. The kinetic models used for analysis were Michaelis–Menten kinetics (eq. 1), biphasic kinetics (eq. 2) and inhibition kinetics (eq. 3)^32^.
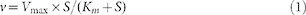

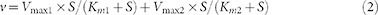

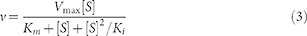
where V_max_ is the maximal velocity and *K*_m_ is the substrate concentration at half- maximal velocity. *K*_si_ is the constant describing the substrate inhibition interaction. All of the incubations were performed in three independent experiments in duplicate. Kinetic constants were obtained using Origin 7.5 (OriginLab Corp., Northampton, MA) and were reported as the mean ± standard error (S.E.) of the parameter estimate.

## Author Contributions

Participated in the research design: M.X.C. Conducted experiments: J.L., W.C., H.X.K. and D.S. Contributed new reagents or analytic tools: H.X.K., L.K.X. and M.X.C. Performed data analysis: M.X.C., J.L., L.S.C., Q.X.Y. and G.G.B. Wrote or contributed to the writing of the manuscript: J.L. and M.X.C. J.L. and L.S.C. contributed equally to this work.

## Supplementary Material

Supplementary Information Supplementary Information

## Figures and Tables

**Figure 1:**
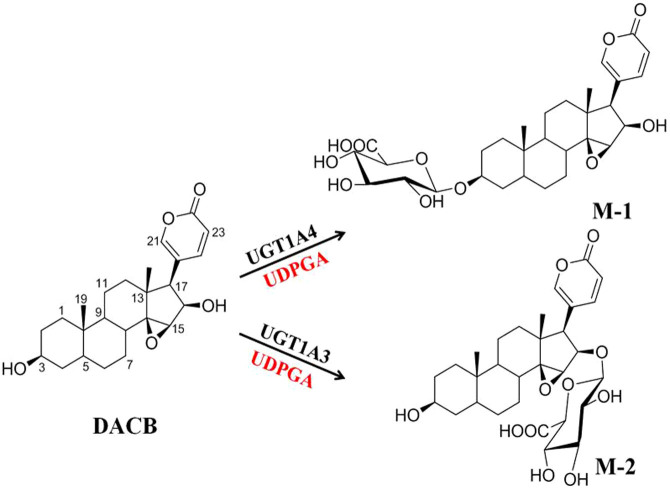
DACB 3*β*- and 16*β*-*O*-glucuronidation by UGT1A4 and UGT1A3, respectively.

**Table 1:** Kinetic parameters of DACB 3-*O*- and 16-*O*-glucuronidation in different enzyme resources

		K_m_	V_max_	K_i_
	Enzymes	μM	nmol/min/mg	μM
**M-1**	**HLMs**	168.18 ± 16.1	0.061 ± 0.002	N/A
	**UGT1A4**	114.84 ± 7.7	0.005 ± 0.001	N/A
**M-2**	**HLMs**	61.29 ± 7.24	0.074 ± 0.004	1290.6 ± 236.7
	**HIMs**	369.36 ± 143.2	0.007 ± 0.002	358.4 ± 157.5
	**UGT1A1**	471.60 ± 93.2	0.012 ± 0.001	N/A
	**UGT1A3**	15.03 ± 2.3	0.307 ± 0.021	332.66 ± 52.1

V_max_ values are expressed in nmol min^−1^ mg^−1^ of protein for HLMs or nmol min^−1^ mg^−1^ of protein for UGTs 1A1, 1A3 and 1A4. The range of substrate concentrations is 0.2 to 800 μM. Each value is the mean ± standard deviation (S.D.) of three determinations performed in duplicate.
