# Supplementary material for: Identifying and applying a highly selective probe to simultaneously determine the O-glucuronidation activity of human UGT1A3 and UGT1A4
Source: Sci Rep. 2015 Apr 17;5:9627. doi: 10.1038/srep09627 (PMC4401096; doi:10.1038/srep09627)
Supplement: Supplementary Information [file srep09627-s1.pdf]

Supplementary Data

Identifying and applying a highly selective probe to simultaneously determine the *O*-glucuronidation activity of human UGT1A3 and UGT1A4

Li Jiang,[1,+] Si-Cheng Liang,[2,3,+] Chao Wang,[1] Guang-Bo Ge,[2] Xiao-Kui Huo,[1] Xiao-Yi Qi,[4] Sa Deng,[1] Ke-Xin Liu [1] and Xiao-Chi Ma[1,*]

[1] College of Pharmacy, Key Laboratory of Pharmacokinetic and Drug Transport of Liaoning, Academy of Integrative Medicine, Dalian Medical University, Dalian, 116044, China.

[2] Laboratory of Pharmaceutical Resource Discovery, Dalian Institute of Chemical Physics, Chinese Academy of Sciences, Dalian, China.

[3] Graduate School of Chinese Academy of Sciences, Beijing, China.

[4] Second Affiliated Hospital of Dalian Medical University, Dalian, China

Correspondence and requests for materials should be addressed to X.C. Ma (E-mail: maxc1978@163.com)

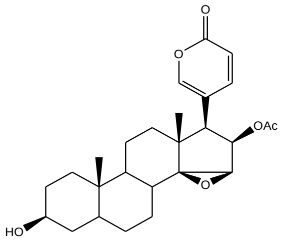

1

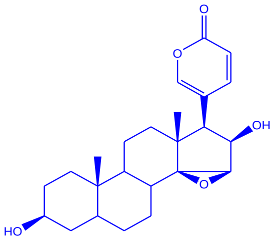

2 (DACB)

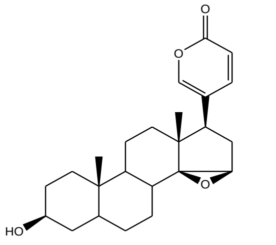

3

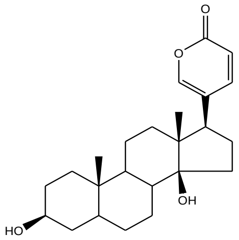

4

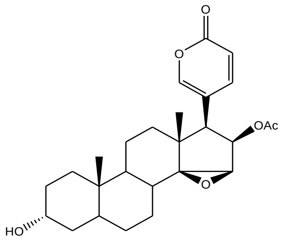

5

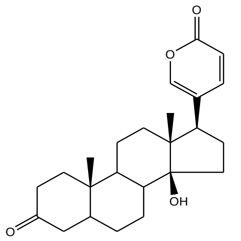

6

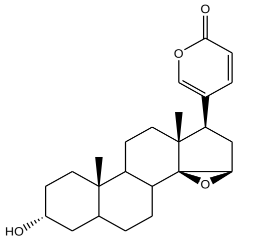

7

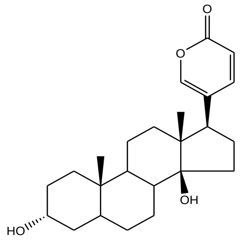

8

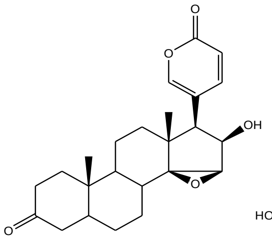

9

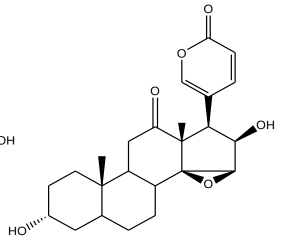

10

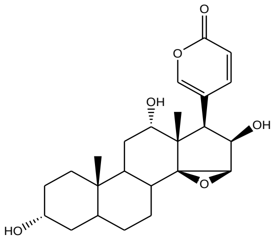

11

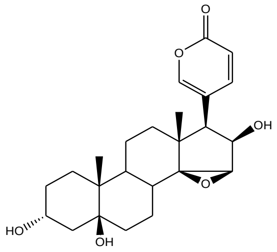

12

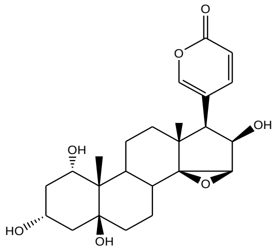

13

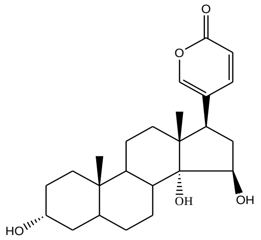

14

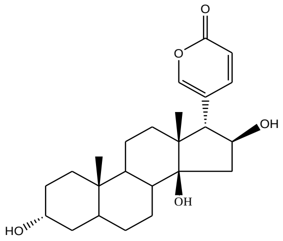

15

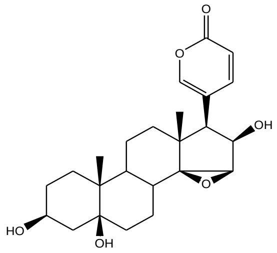

16

Fig. S1 Chemical structures of bufadienolides (**1-16**) used in the present work

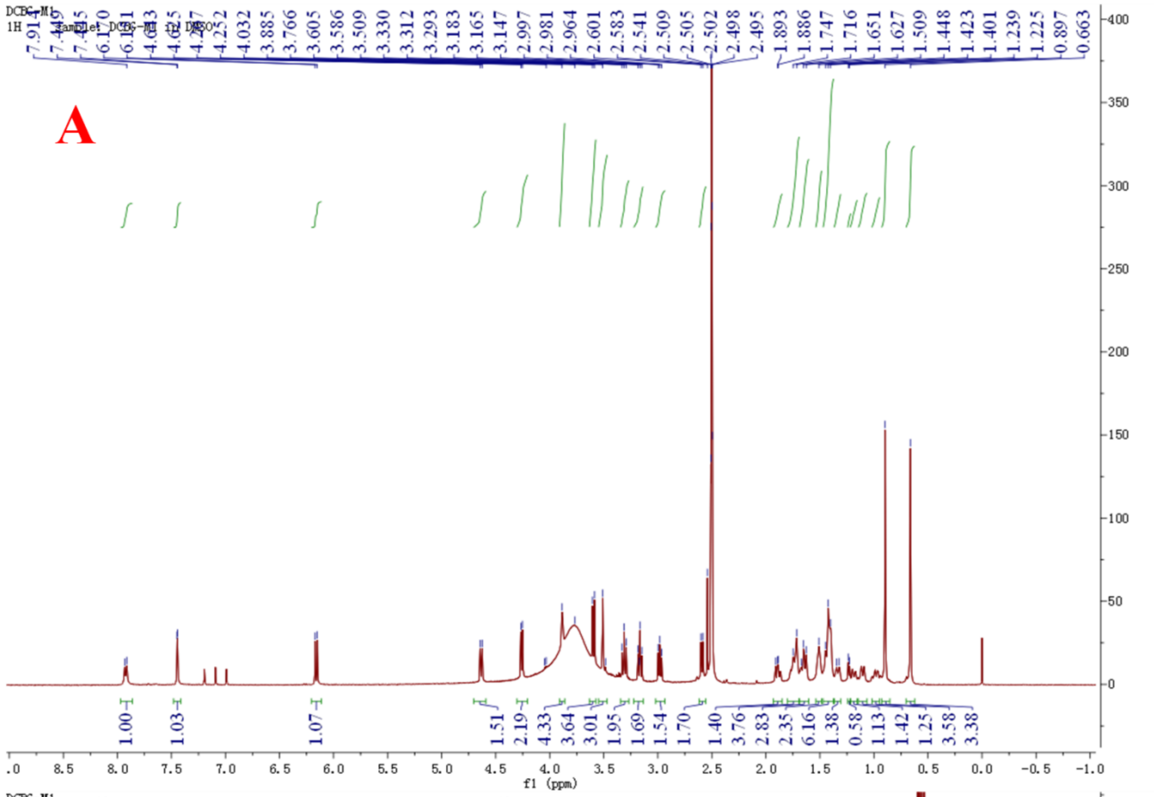

A
f1 (ppm)

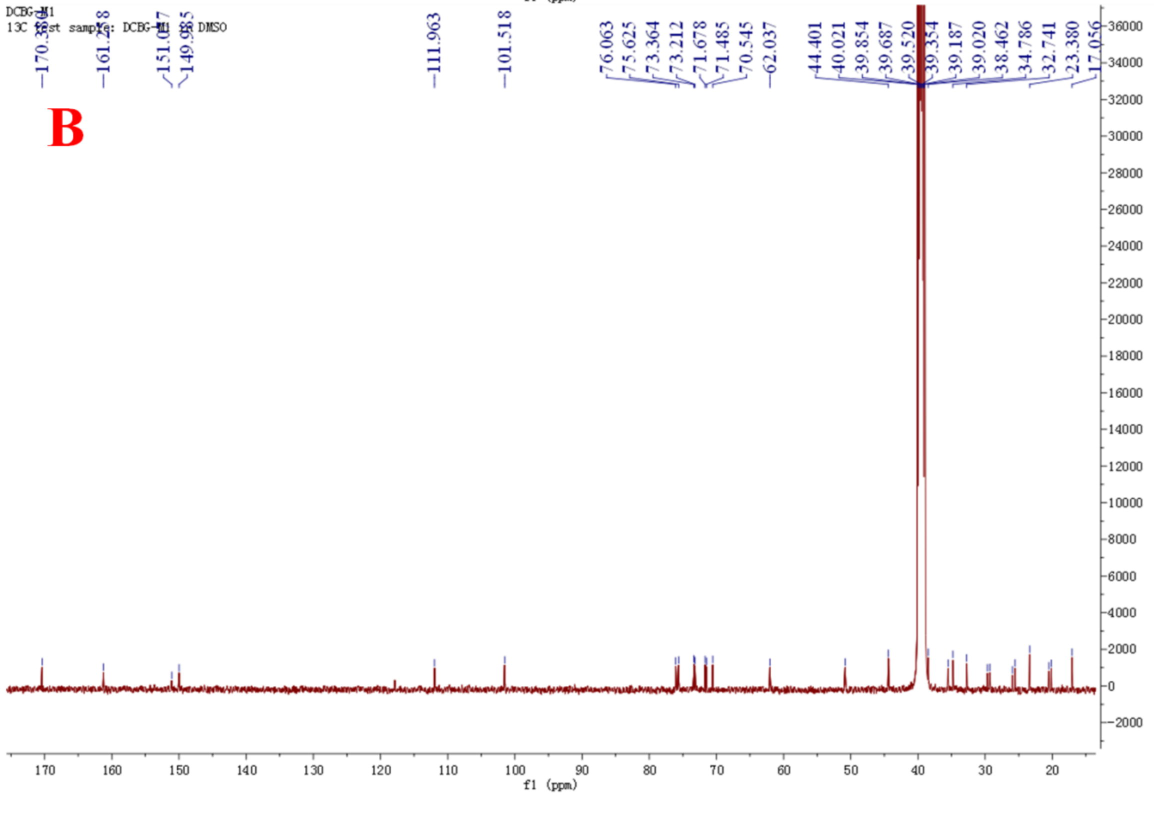

B
f1 (ppm)

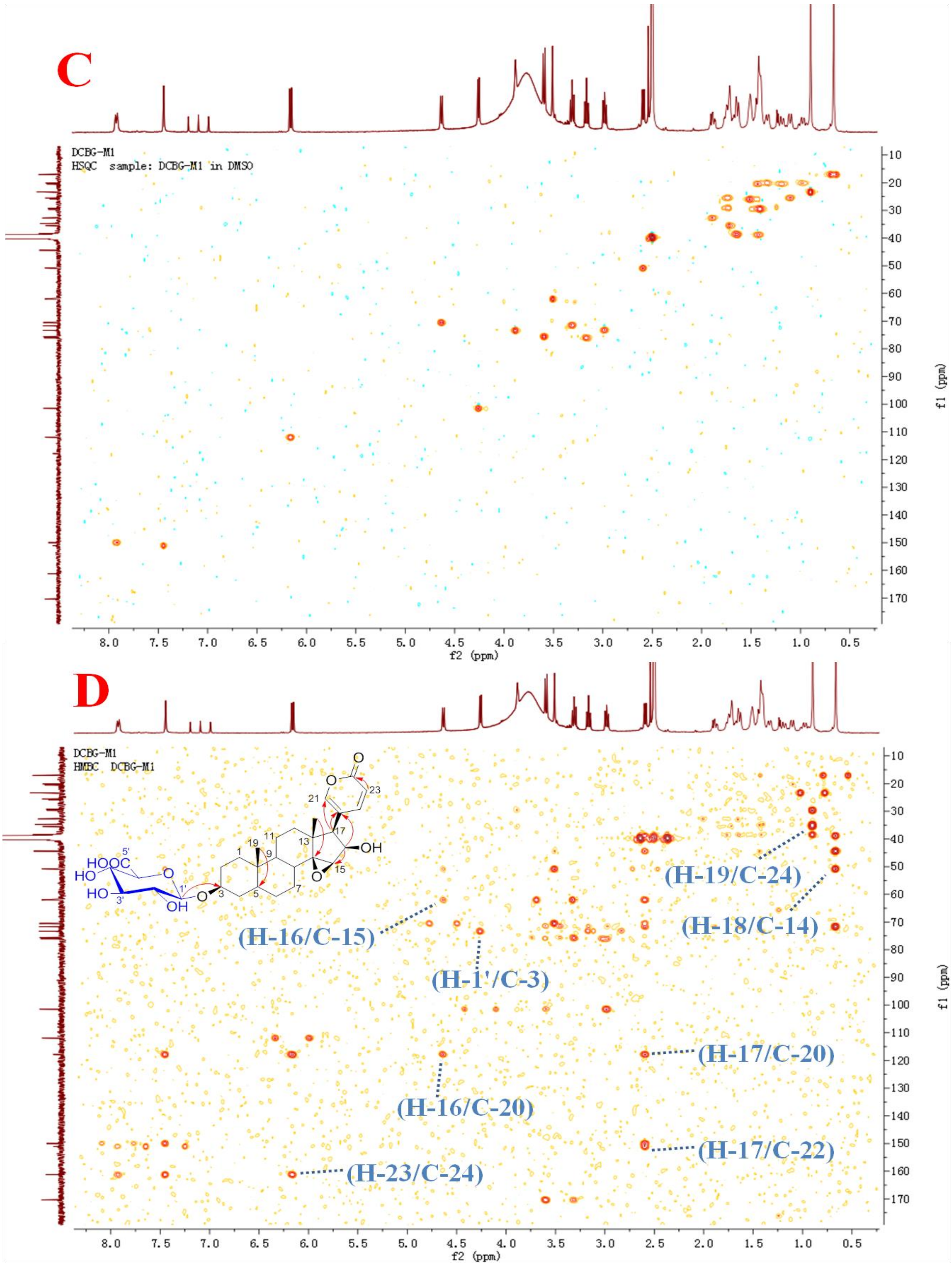

Fig. S2. The spectral data of M-1 (500MHz, DMSO). ^{1}H-NMR (A); ^{13}C-NMR (B); HMQC (C) and HMBC (D).

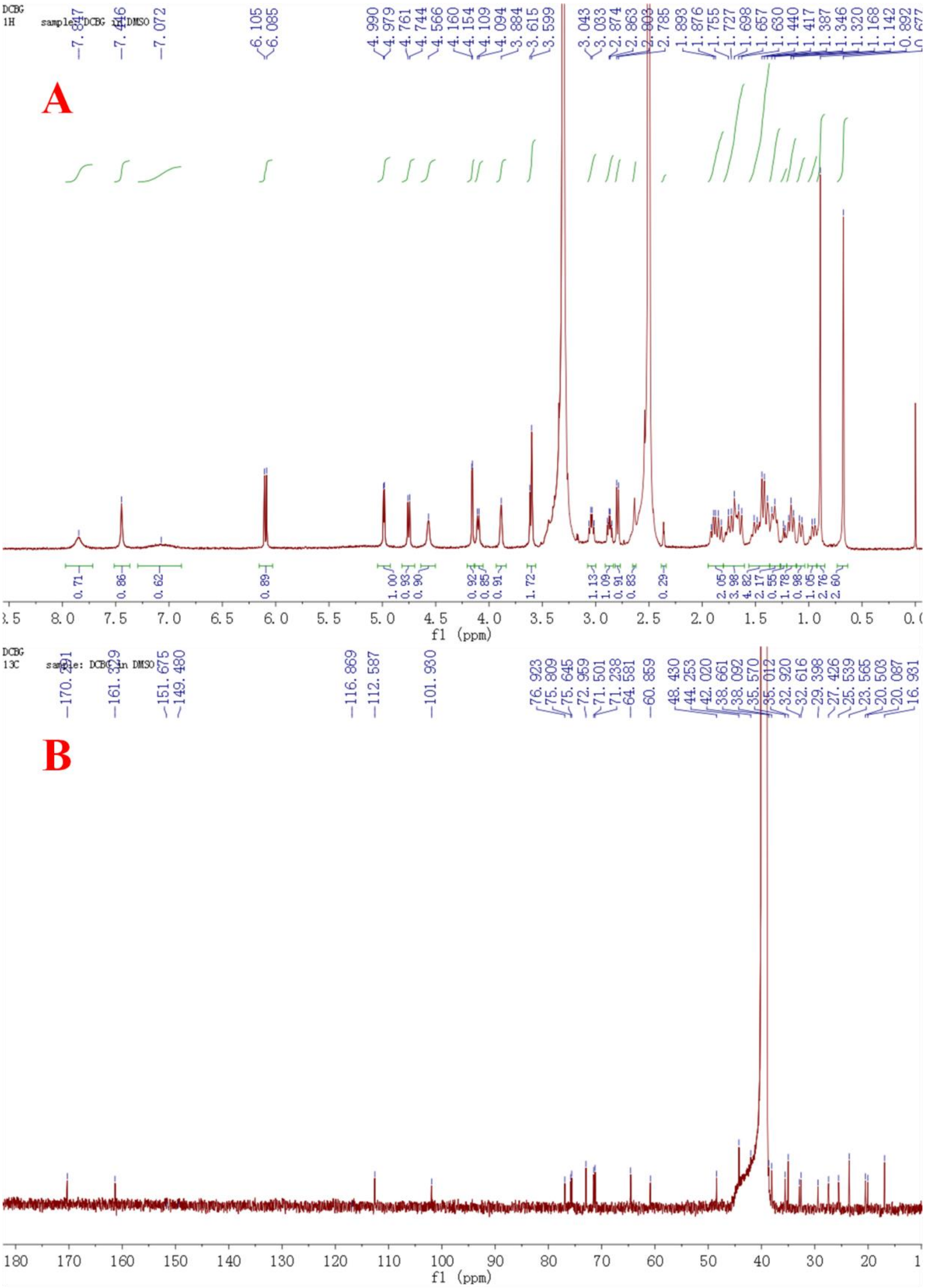
DCBG
1H sample: DCBG in DMSO
A
6.105
6.085
4.990
4.979
4.761
4.744
4.566
4.160
4.154
4.109
4.094
3.884
3.615
3.599
3.043
3.033
2.874
2.863
2.785
1.893
1.876
1.755
1.727
1.698
1.657
1.630
1.440
1.417
1.387
1.346
1.320
1.168
1.142
0.892
f1 (ppm)
DCBG
13C sample: DCBG in DMSO
B
151.675
149.480
116.869
112.587
101.930
76.923
75.809
75.645
72.959
71.501
71.238
64.581
60.859
48.430
44.253
42.020
38.661
38.092
35.570
35.012
32.920
32.616
29.398
27.426
25.539
23.565
20.503
20.087
16.931
f1 (ppm)

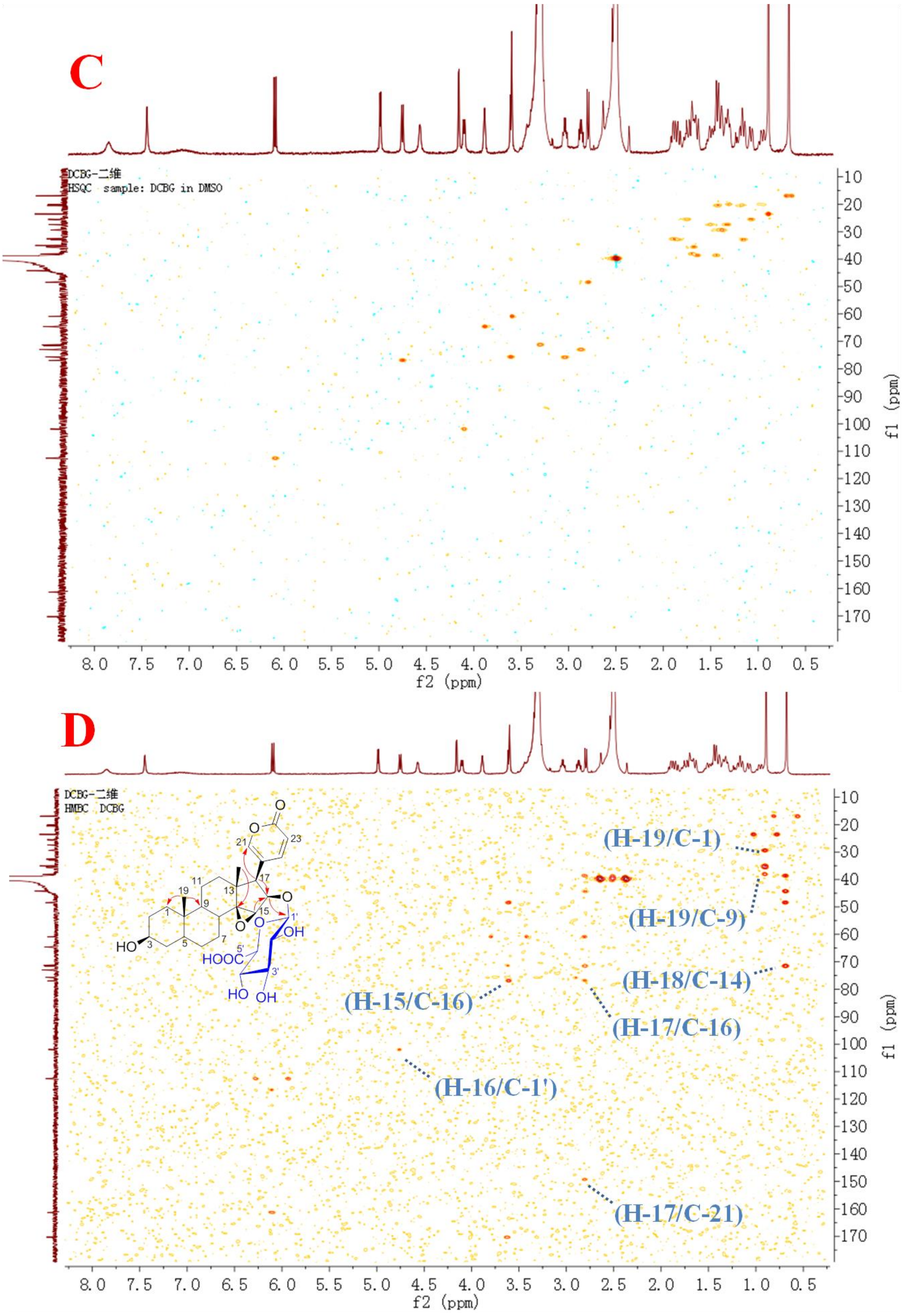

Fig. S3. The spectral data of M-2 (500MHz, DMSO). ^{1}H-NMR (A); ^{13}C-NMR (B); HMQC (C) and HMBC (D).

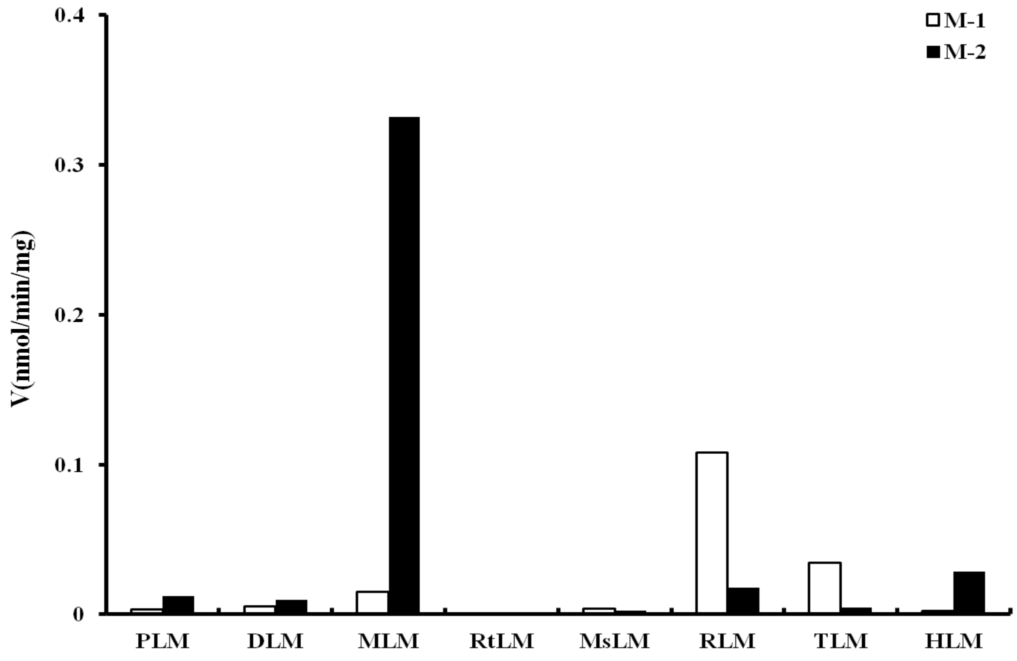

Fig. S4 The formation rates of M-1 and M-2 in various species, including PLMs, DLMs, MLMs, RtLMs, MsLMs, RLMs, TLMs and HLMs.

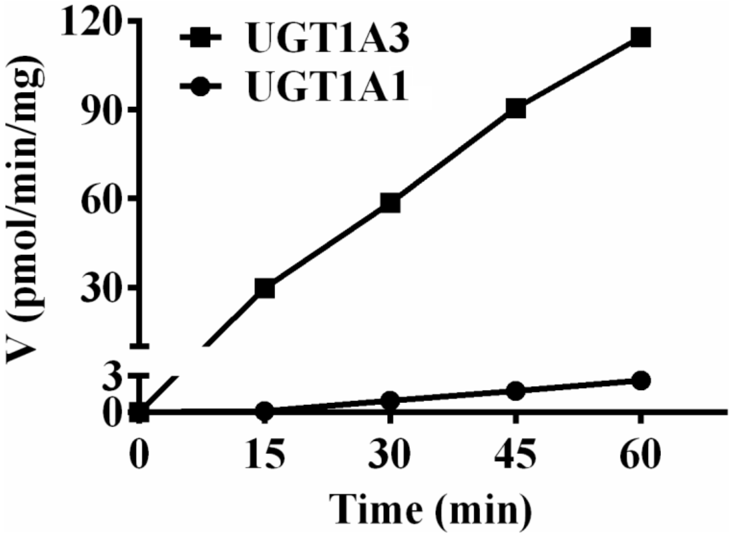

Fig. S5 Time courses for DACB-16-*O*-glucuronidation by UGT1A3 and UGT1A1. The final substrate concentration was 50 μM. Each data point represents the mean of triplicate determinations.

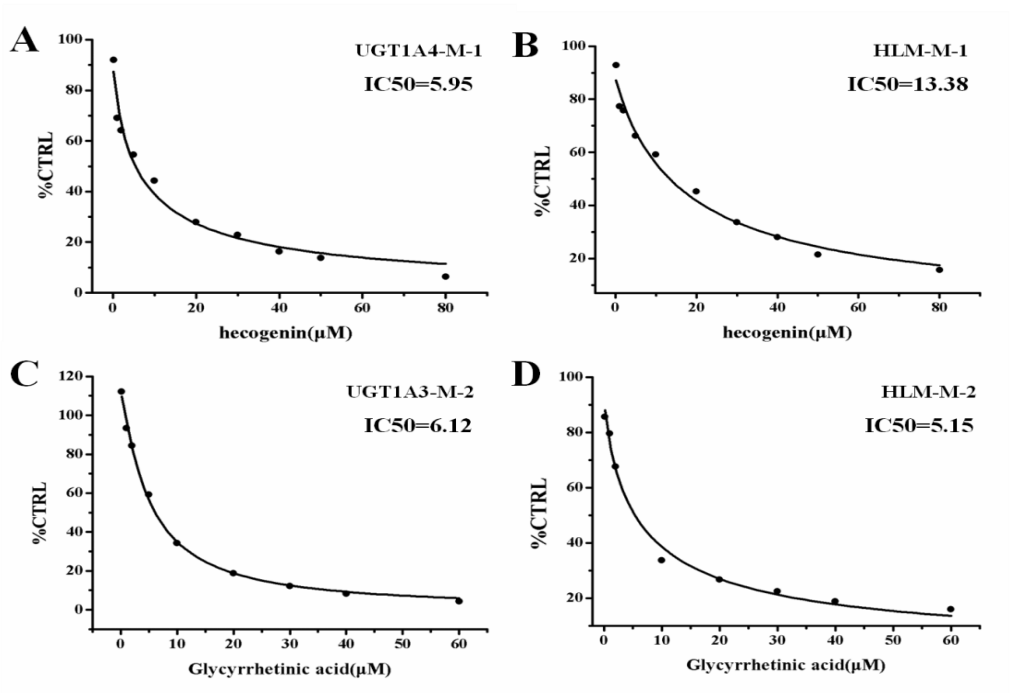

Fig. S6. IC_{50} values of M-1 formation by hecogenin (a UGT1A4-specific inhibitor) in UGT1A4 samples (A) and HLMs (B); and IC_{50} values for M-2 formation by glycyrrhetinic acid (a UGT1A3-specific inhibitor) in UGT1A3 samples (C) and HLMs (D), respectively.

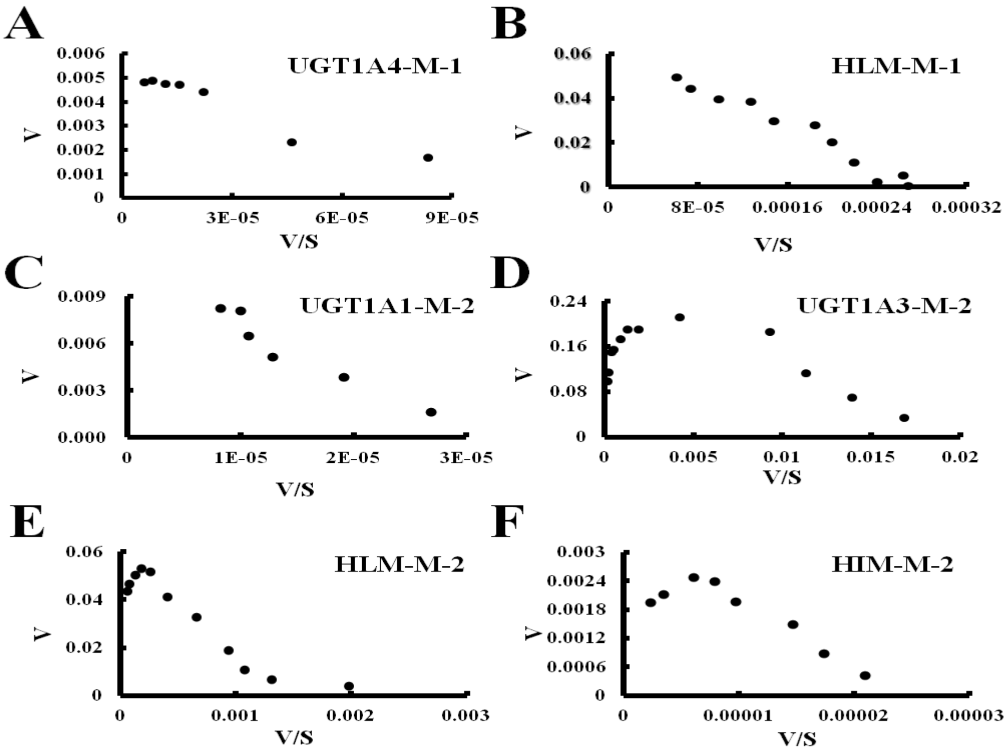

Fig. S7 Eadie-Hofstee plots of the glucuronidation profiles to determine the best-fit equation. **(A)** UGT1A4 samples for M-1; **(B)** HLMs for M-1; **(C)** UGT1A1 samples for M-2; **(D)** UGT1A3 samples for M-2; **(E)** HLMs for M-2; and **(F)** HIMs for M-2.

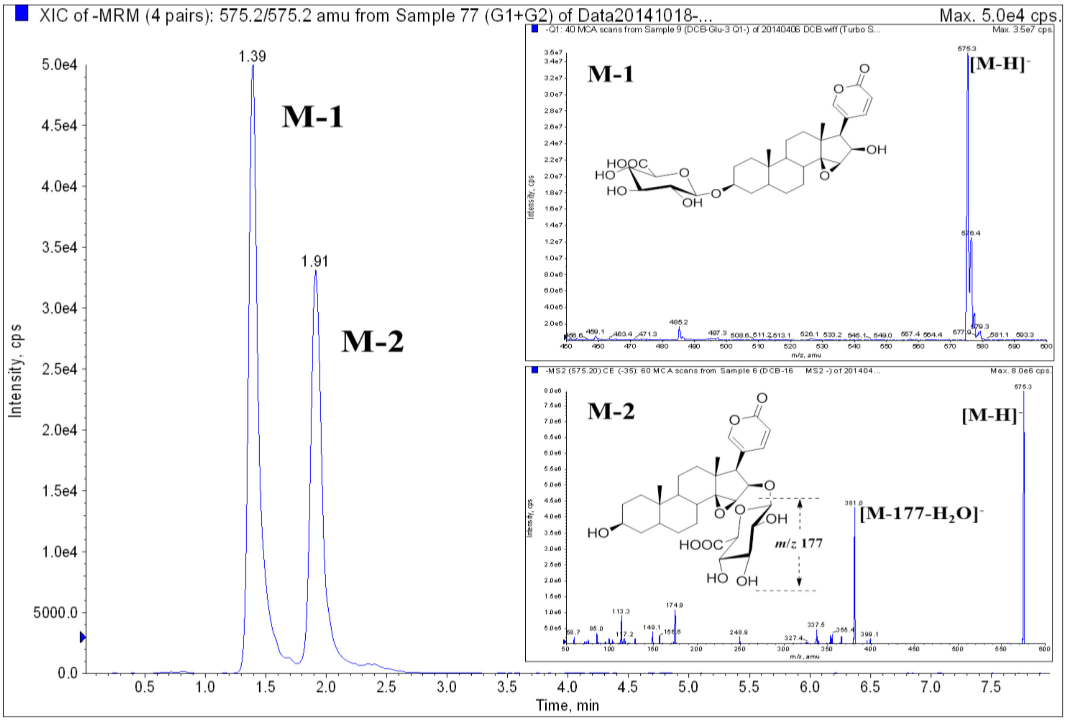

Fig. S8 MRM chromatogram of M-1 and M-2 in HLMs.

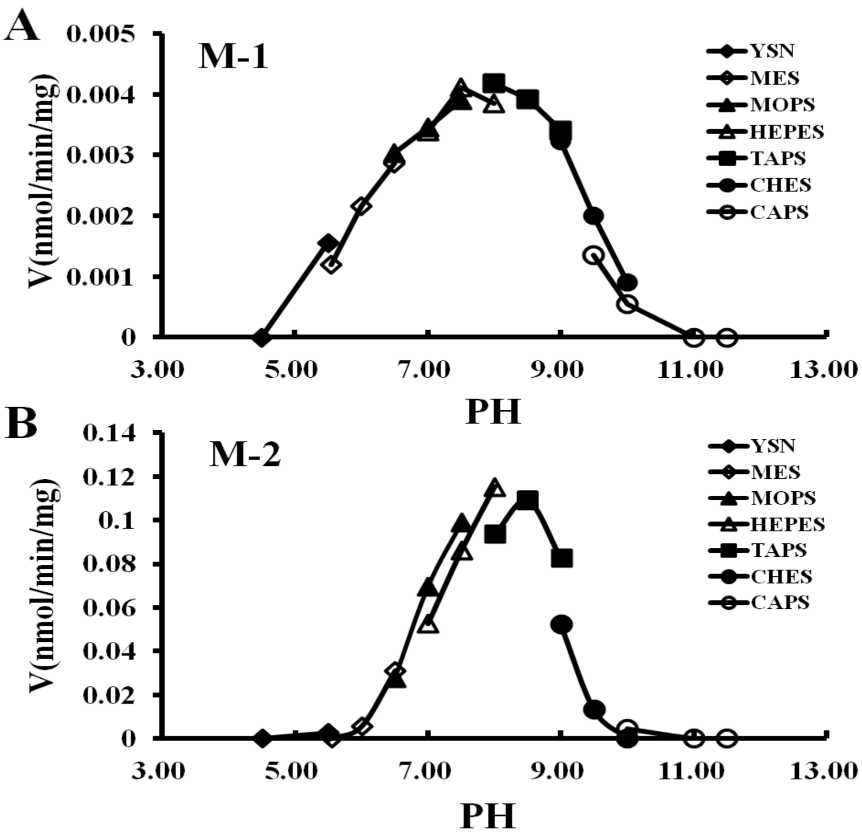

Fig. S9 The influences of various pH values for forming M-1(A) and M-2 (B).

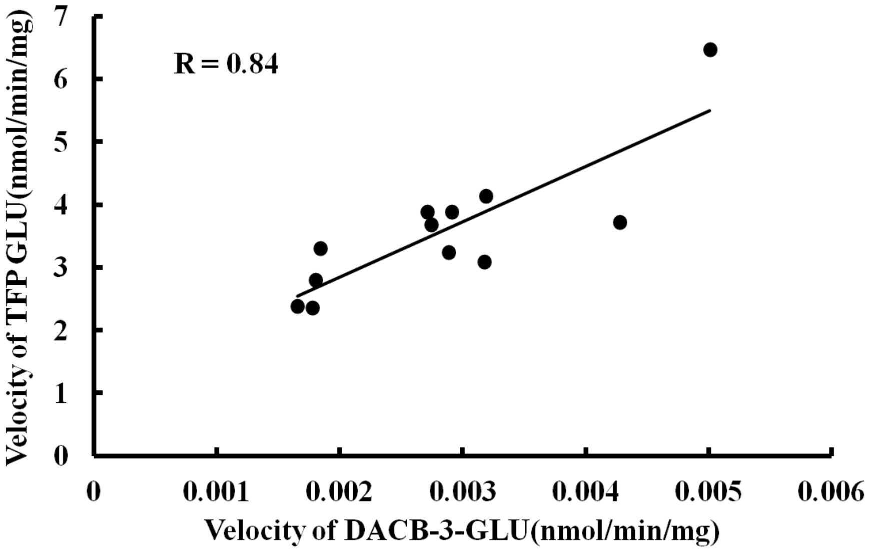

Fig. S10 The correlation analysis between DACB-3-*O*-glucurondiation rates by UGT1A4 and TFP-*N*-glucuronidation by UGT1A4 in 12 individual HLMs.

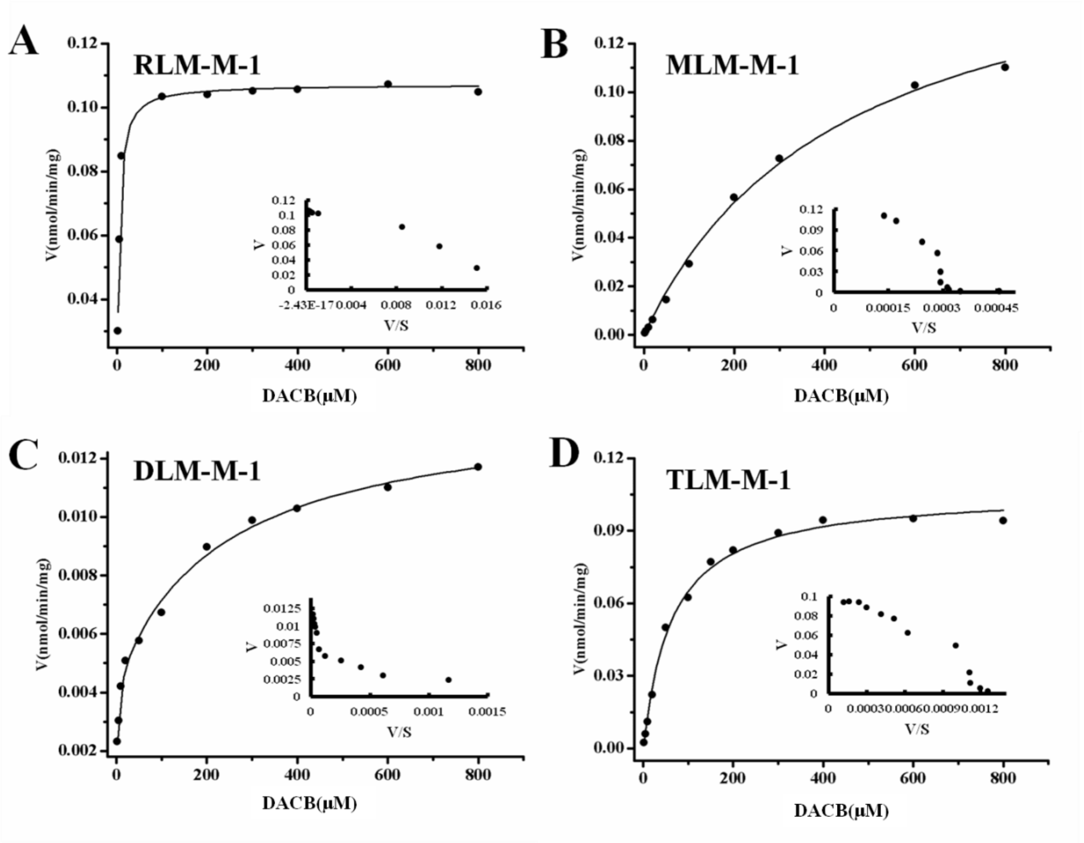

Fig. S11 The enzyme kinetics of DACB glucuronidation at C-3 (M-1) in animal liver microsomes. (A) Rabbit liver microsomes; (B) Monkey liver microsomes; (C) Dog liver microsomes and (D) Guinea pig liver microsomes.

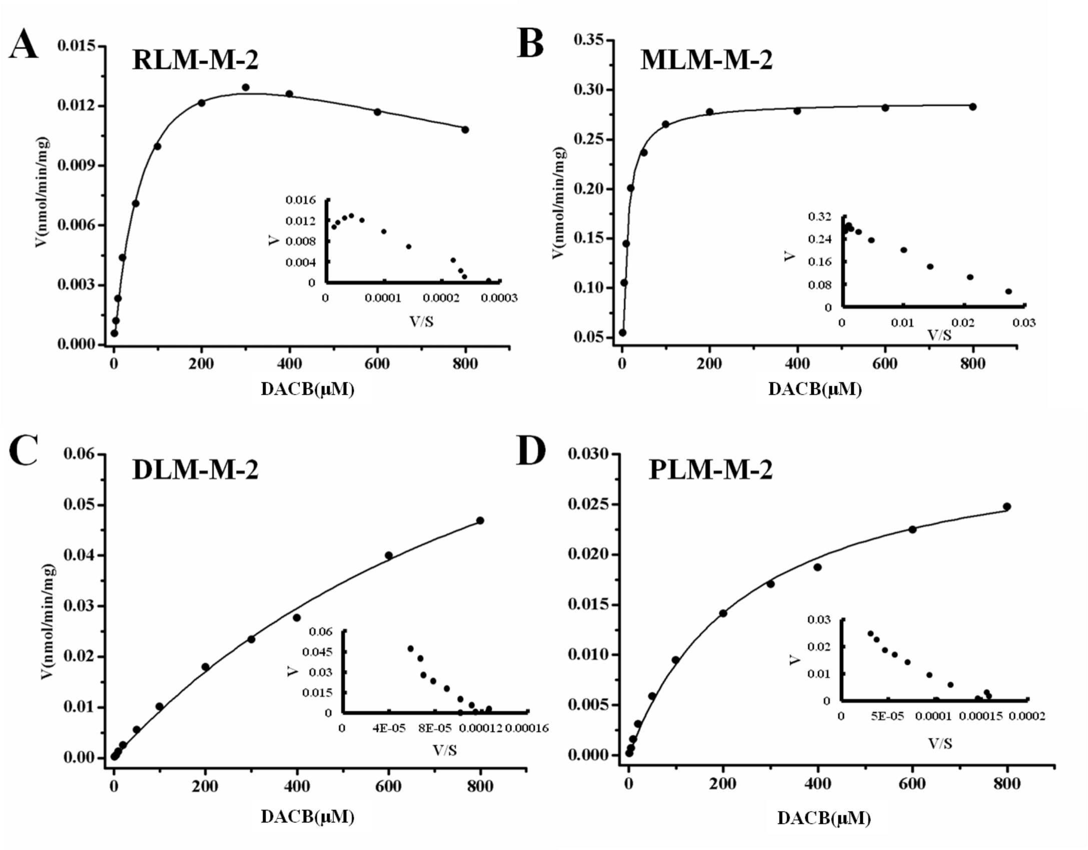

Fig. S12 The enzyme kinetics of DACB glucuronidation at C-16 (M-2) in animal liver microsomes. (A) Rabbit liver microsomes; (B) Monkey liver microsomes; (C) Dog liver microsomes and (D) Pig liver microsomes.

Table S1 ^{1}H- and ^{13}C NMR spectral data of M-1 and M-2 (DMSO-d_6)

No.	M-1		M-2	
	δ_H (500 MHz)	δ_C (125 MHz)	δ_H (500 MHz)	δ_C (125 MHz)
**1**	1.72 m<br>1.41 m	29.3	1.43 m<br>1.38 m	29.4
**2**	1.51 m<br>1.44 m	25.9	1.50 m<br>1.32 m	27.4
**3**	3.89 brs	73.4	3.88 s	64.6
**4**	1.50 m<br>1.41 m	29.7	1.84 m<br>1.15 m	32.6
**5**	1.72 m	35.5	1.70 m	35.6
**6**	1.74 m<br>1.11 m	25.5	1.75 m<br>1.07 m	25.5
**7**	1.44 m<br>1.19 m	20.5	1.43 m<br>1.17 m	20.5
**8**	1.89 m	32.7	1.87 m	32.9
**9**	1.65 m	38.5	1.70 m	38.1
**10**	—	34.8	—	35.0
**11**	1.34 m<br>0.98 m	20.2	1.31 m<br>0.95 m	20.1
**12**	1.65 m<br>1.43 m	38.7	1.64 m<br>1.44 m	38.7
**13**	—	44.4	—	44.3
**14**	—	71.7	—	71.5
**15**	3.51 brs	62.0	3.60 s	60.9
**16**	4.63 d (9.0)	70.5	4.79 brd (8.5)	76.9
**17**	2.59 d (9.0)	50.9	2.79 d (8.5)	48.4
**18**	0.66 s	17.1	0.68 s	16.9
**19**	0.90 s	23.4	0.89 s	23.6
**20**	—	117.9	—	116.6
**21**	7.45 d (2.0)	151.1	7.40 s	151.8
**22**	7.92 d (9.5)	150.0	7.80 brs	149.6
**23**	6.10 d (9.5)	112.0	6.10 d (10.0)	112.6
**24**	—	161.3	—	161.3
**1'**	4.26 d (7.5)	101.5	4.10 d (7.5)	101.9
**2'**	3.31 t (9.0)	71.5	3.30 m	71.2
**3'**	3.17 t (9.0)	76.1	3.04 m	75.9
**4'**	2.98 t (9.0)	73.2	2.87 m	73.0
**5'**	3.60 d (9.0)	75.6	3.61 d (8.0)	75.6
**6'**	—	170.4	—	170.3

Table S2 Kinetic parameters of DACB-*O*-glucuronidation in microsomes of experimental animals

**Metabolites**	**Enzymes**	**K_m**	**V_{max}**	**Cl_{int}**	**K_i**
		μM	nmol/min/mg	μL/min/mg	μM
**3-*O*-Glucuronide (M-1)**	**RLMs**	3.95±0.41	0.107±0.002	27.848	N/A
	**MLMs**	439.53±40.19	0.174±0.008	0.387	N/A
	**DLMs**	2.19±0.76	0.005±0.001	2.054	N/A
		241.13±63.40	0.009±0.001	0.038	N/A
	**TLMs**	63.58±5.33	0.106±0.002	1.730	N/A
**16-*O*-Glucuronide (M-2)**	**RLMs**	75.43±7.01	0.018±0.001	0.239	1284.5±187.2
	**MLMs**	9.18±0.41	0.288±0.002	31.59	N/A
	**DLMs**	1094.8±140.1	0.111±0.010	0.100	N/A
	**PLMs**	251.6±22.7	0.032±0.001	0.127	N/A
